# The Essential Oil from the Resurrection Plant *Myrothamnus moschatus* Is Effective against Arthropods of Agricultural and Medical Interest

**DOI:** 10.3390/ph15121511

**Published:** 2022-12-03

**Authors:** Roman Pavela, Marta Ferrati, Eleonora Spinozzi, Filippo Maggi, Riccardo Petrelli, Rianasoambolanoro Rakotosaona, Renato Ricciardi, Giovanni Benelli

**Affiliations:** 1 Crop Research Institute, Drnovska 507, 161 06 Prague, Czech Republic; 2 Department of Plant Protection, Czech University of Life Sciences Prague, Kamycka 129, 165 00 Praha 6, Czech Republic; 3 Chemistry Interdisciplinary Project (ChIP), School of Pharmacy, University of Camerino, 62032 Camerino, Italy; 4 Centre National d’Application de Recherches Pharmaceutiques, Antananarivo 101, Madagascar; 5 Department of Agriculture, Food and Environment, University of Pisa, via del Borghetto 80, 56124 Pisa, Italy

**Keywords:** eco-friendly insecticide, sustainable acaricide, *Myrothamnus moschatus*, *Metopolophium dirhodum*, *Spodoptera littoralis*, *Tetranychus urticae*, *Culex quinquefasciatus*, *Musca domestica*

## Abstract

This work aimed to evaluate the chemical composition, insecticidal and acaricidal potential of the essential oil (EO) obtained from the resurrection plant *Myrothamnus moschatus* (Baill.) Baill. (Myrothamnaceae) from Madagascar. The EO bioactivity was evaluated against selected arthropod pests and vectors of agricultural and public health relevance. The most abundant volatile compounds were *trans*-pinocarveol (37.7 ± 4.2%) and pinocarvone (20.8 ± 3.1%), similar to the EO of the chemotype collected from the same region. Lethal concentrations (LC_50_) or doses (LD_50_) from acute toxicity tests were estimated for *Musca domestica* (L.) adults at 22.7 µg adult^−1^, for *Spodoptera littoralis* (Boisduval) larvae at 35.6 µg larva^−1^, for *Culex quinquefasciatus* (Say) at 43.6 µg mL^−1^, for adults of *Metopolophium dirhodum* (Walker) at 2.4 mL L^−1^, and for adults of *Tetranychus urticae* (Koch) at 1.2 mL L^−1^. The good insecticidal and acaricidal activities determined in this work may open a new perspective on the use of this plant as a source of botanical insecticide ingredients. The exploitation of this species could also be important for the African economy, helping local farmers cultivating this plant.

## 1. Introduction

In the last century, the world population has risen dramatically, and it is estimated that by 2050 food production will have to increase by 70% [1]. To guarantee a wide crop’s effectiveness, pesticides play a fundamental role. They can reduce the development of several plant diseases and ensure the recovery of 30–40% of crop losses worldwide [2,3]. However, conventional chemical pesticides can be toxic to other beneficial organisms and non-target plants, as well as to air, water, and soil, resulting in environmental pollution and a threat to human health [2]. Moreover, long-lasting uses of pesticides can often lead to resistance phenomena in the targeted arthropod populations [4].

For the above reason, there has been a growing global interest in eco-friendly pesticides, including those produced by plants [5,6,7]. Among botanical products, essential oils (EOs) could play a fundamental role in the agrochemical industries, and they have been widely studied against many arthropod pests [8,9,10,11]. EOs showed different modes of action, such as antifeedant, repellent, or deterrent activities, and they can be useful as larvicide, ovicide, and adulticidal agents. Their main constituents could act at different stages of insect and mite development [12]. Moreover, EOs are often made up of several active substances with multiple modes of action that avoid the development of resistance phenomena in targeted pests [13].

**Figure 1:**
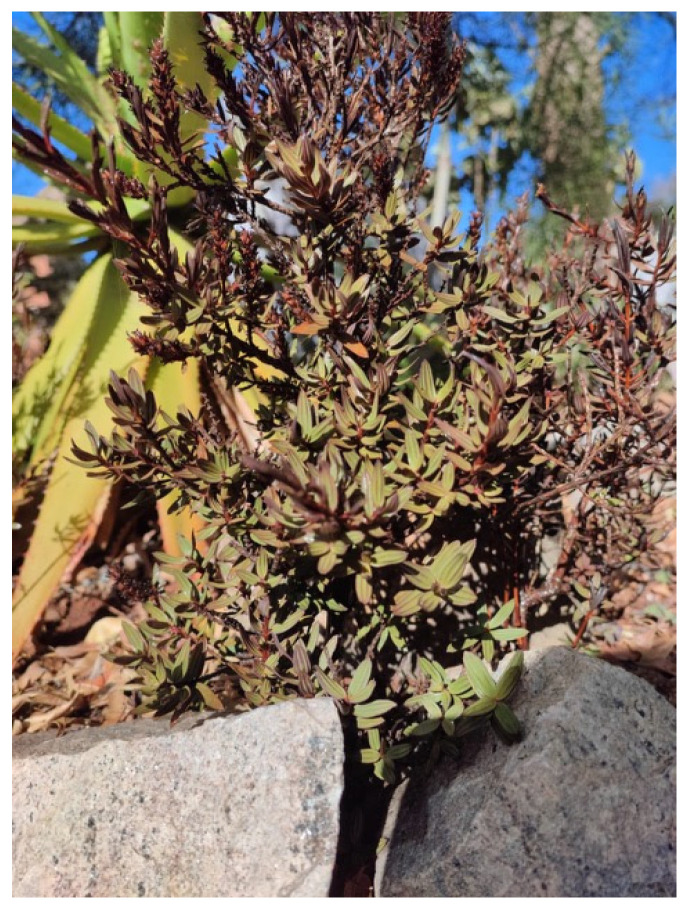
*Myrtothamnus moschatus* during the rainy season.

*Myrothamnus moschatus* (Baill.) Baill. is a small dioecious shrub, endemic in Madagascar and belonging to the Myrothamnaceae family [14]. It belongs to a group of plants containing more than 1300 species that are called ‘resurrection plants’, owing to their remarkable peculiarity of being able to desiccate in a drought state and restart the photosynthetic activity once the stressed condition ends (Figure 1). Therefore, they can remain quiescent for long periods of time, ‘resurrecting’ at the first substantial fall of rain [15]. *Myrothamnus moschatus* has been used for many purposes, from medicinal to recreational. For instance, Malagasy people used to smoke the dried leaves to treat asthma and for their psychoactive action reminiscent of that of marijuana [16,17]. The plant is also used as an anti-emetic and to treat coughs [18], while in the central highlands, the leaves are burned during magical rituals to expel the devil [19].

In the past, ‘resurrection’ plants were mainly studied for their ability to remain quiescent under stressed conditions [20,21,22]. However, recently, interest in these plants has shifted towards the potential of their products, such as extracts and EOs. They contain many compounds that demonstrate a wide range of properties in experimental models [23,24,25]. The biological activities of the EO of *M. moschatus* were investigated for the first time in 2012 by Nicoletti et al. [18]. To date, this EO has shown a strong inhibition potential toward cancerous cells, as well as antifungal, anticonvulsant, and antioxidant activities [18,26]. However, there are still relatively few studies on *M. moschatus* EO, and, to the best of our knowledge, there are no data available on the insecticidal potential of this plant. 

Therefore, the aim of this work was to assess, for the first time, the effectiveness of this plant EO against some noxious arthropod pests and vectors of agricultural and public health relevance, i.e., the rose grain aphid, *Metopolophium dirhodum* (Walker) (Hemiptera: Aphididae), the cotton leafworm, *Spodoptera littoralis* (Boisduval) (Lepidoptera: Noctuidae), and the two-spotted mite, *Tetranychus urticae* (Koch) (Acari: Tetranychidae), as well as representative insect species of urban importance. i.e., the mosquito vector *Culex quinquefasciatus* (Say) (Diptera: Culicidae) and the common house fly, *Musca domestica* (L.) (Diptera: Muscidae).

## 2. Results and Discussion

The chemical variability of the *M. moschatus* EO observed in this study comprises 34 compounds, representing 98.2% of the total oil composition. The results obtained by GC-MS analysis are shown in Table 1. Most of the constituents are oxygenated monoterpenes (75.3%), while monoterpene hydrocarbons (12.6%) and sesquiterpene hydrocarbons (10.2%) are minor chemical classes. The most abundant volatile compounds were *trans*-pinocarveol (37.7 ± 4.2%) and pinocarvone (20.8 ± 3.1%), followed by *β*-selinene (10.2 ± 1.3%), *α*-pinene (8.8 ± 1.2%), and perillyl acetate (4.9 ± 0.9 %). Myrtenal (1.8 ± 0.3%), *p*-cymene (1.8 ± 0.4%), myrtenol (1.5 ± 0.3), *trans*-*p*-mentha-1(7),8-dien-2-ol (2.2 ± 0.4%), and *cis*-*p*-mentha-1(7),8-dien-2-ol (1.4 ± 0.3%) were detected in minor percentages.

*Myrothamnus moschatus* EO has been studied for its chemical variability among species of different geographical origins. It was demonstrated that the occurrence of different chemotypes depends on the plant variability, but also on the interaction with the environment and genetic selection [27]. The chemical composition of this EO is very similar to those obtained by *M. moschatus* plants harvested in the same region in previous studies [18,19]. Even if these latter were collected in different seasons, it was demonstrated that the environmental conditions do not influence the EO chemical composition of this species [18]. Conversely, Randrianarivo et al., [28] analyzed the EOs of *M. moschatus* plants growing in different areas of Madagascar. They showed many differences, depending on the plant variability, but also on the environmental parameters and genetic selection. As a confirmation, the chemotype of this work was widely different from those of plants collected in other regions. EOs of the plants harvested in the central-northern part of the island were mainly composed of oxygenated sesquiterpenes. Conversely, southern populations were mainly constituted of oxygenated monoterpenes. In particular, the chemotype of the Ifandana populations was quite comparable to that of our study [28].

Comparing our results with those reported for the other species of the genus *Myrothamnus*, i.e., *M. flabellifolia* Welw., we observed a similar profile, with *trans*-pinocarveol, pinocarvone, limonene, *trans*-*p*-mentha-1(7),8-dien-2-ol, *cis*-*p*-mentha-1(7),8-dien-2-ol, *α*-pinene, and *β*-selinene as the main EO component of this species [29,30]. It is worth noting that the monoterpenoid perillyl acetate, an uncommon EO constituent, was detected only in the *M. moschatus* species. This compound has been demonstrated to prevent carcinoma of the breast in rats [31]. The other major compounds, *trans*-pinocarveol and its oxidation product, pinocarvone, have been correlated to the ethnobotanical use of the plant [19]. The former seems effective against coughs, being included in pharmaceutical preparations to treat respiratory tract diseases [30], while the latter appears effective against colds [32]. These molecules are also released when the plant is smoked by Malagasy people during magic rituals [30].

To the best of our knowledge, no data are available in the literature on the insecticidal and acaricidal properties of this EO and their components. Therefore, this work represents the first investigation of its effectiveness against arthropod vectors and pests, with the aim of widening the possible application of this plant in Madagascar. The EO analyzed here showed relatively good insecticidal activity, and this EO and its main components, whenever available as chemical reagents, can be considered promising active ingredients of new botanical insecticides and acaricides. EO lethal concentrations (LC_50_) or doses (LD_50_) from acute toxicity tests (Table 2) were estimated for *M. domestica* adults at 22.7 µg adult^−1^, for *S. littoralis* larvae at 35.6 µg larva^−1^, for *Cx. quinquefasciatus* at 43.6 µg mL^−1^, for adults of *M. dirhodum* at 2.4 mL L^−1^, and for adults of *T. urticae* at 1.2 mL L^−1^. Testing a positive control is an important requirement for validating green insecticide research [33,34,35]. Table 3 reports the acute toxicity results achieved testing a positive control, i.e., a commercial botanical insecticide based on *Pongamia pinnata* L. (Pierre) oil, commercially known as Rock Effect (Agro CS a.s., Česká Skalice, Czech Republic) on the same arthropod targets. Lethal concentrations (LC_50_) or doses (LD_50_) from acute toxicity tests (Table 2) were estimated for *M. domestica* adults at >500 µg adult^−1^, for *S. littoralis* larvae at 18.2 µg larva^−1^, for *Cx. quinquefasciatus* at 275.4 µg mL^−1^, for adults of *M. dirhodum* at 12.5 mL L^−1^ and for adults of *T. urticae* at 5.8 mL L^−1^. Comparing *M. moschatus* EO (Table 2) against the positive control (Table 3) highlighted differences in efficacy depending on the target species. The LC_50_ of *M. moschatus* EO in particular was lower when tested on *M. domestica* adults, *Cx. quinquefasciatus* larvae, *M. dirhodum* larvae, and *T. urticae adults*, suggesting greater efficacy than the positive control. These promising results support the development of increasingly effective botanical insecticides for the management of key insect and mite pests.

It should be noted that aphids are generally sensitive to preparations based on EOs. Several botanical insecticides are currently produced commercially against a range of pests, including aphids [36]; for example, a US company, EcoSMART Technologies, produces a ‘Garden Insect Killer’ against sucking pests containing mint and rosemary EO, which is contained in an applied spray liquid in a total dose of 10 mL L^−1^. For example, for EO from *Rosmarinus officinalis* L., the lethal concentration for *Lipaphis pseudo-brassicae* Davis was estimated at 7.4 mL L^−1^ and for *Macrosiphum rosae* (L.) at 57.5 mL L^−1^ [8]. Our estimated lethal concentrations for *M. dirhodum* and *T. urticae* were significantly lower, indicating a promising perspective for the commercial use of *M. moschatus* EO. Similarly, in our work, the lethal concentration estimated for *Cx. quinquefasciatus* larvae was lower than 100 µg mL^−1^, which is generally considered to be the limit of prospective EOs usable mosquito larval management [37]. Of note, our knowledge about the modes of action of the EO’s two major constituents, *trans*-pinocarveol and pinocarvone, is limited. Earlier research showed that these compounds were toxic to various insect species [38,39,40,41,42,43]. For example, Seo et al. [38] demonstrated that *trans*-pinocarveol, used through a fumigant treatment, exhibited a strong fumigant toxicity at all concentrations (0.312, 0.625, 1.25, 2.5, 5, and 10 mg L^−1^) against the Japanese termite, *Reticulitermes speratus* Kolbe (Isoptera: Rhinotermitidae), killing all the exposed individuals; *trans*-pinocarveol at 1 mg mL^−1^ on *R. speratus* adults had an acetylcholinesterase (AChE) inhibition rate of between 15 and 20% [38]. *trans*-Pinocarveol was found among the main components of several EOs, such as that of *Salvia tomentosa* Mill., *Eucalyptus kruseana* Muel, *Croton tetradenius* (Baill), and *Artemisia anethoides* (Mattf.) [39,40,41,42,43]. *Salvia tomentosa* EO tested on stored product beetles i.e., *Acanthoscelides obtectus* (Say) (Coleoptera: Bruchidae), and *Tribolium castaneum* (Herbst) (Coleoptera: Tenebrionidae), *E. kruseana* EO tested on *Rhyzopertha dominica* F. (Coleoptera: Bostrichidae), *C. tetradenius* EO tested on *Acromyrmex balzani* (Emery) (Hymenoptera: Formicidae), and *A. anethoides* EO tested on *T. castaneum* and *Lasioderma serricorne* (Fabricius) (Coleoptera: Anobiidae) achieved good efficacy (*S. tomentosa* EO against *T. castaneum*: LC_50_ = 111.67 µL L^−1^ air, LC_95_ = 174.66 µL L^−1^ air; *S. tomentosa* EO against *A. obtectus*: LC_50_ = 22.47 µL L^−1^ air, LC_95_ = 41.73 µL L^−1^ air; *R. dominica* EO against *E. kruseana*: LC_50_ = 22.98 µL L^−1^ air, LC_90_ = 65.32 µL L^−1^ air; *C. tetradenius* EO against *A. balzani* showed LC_50_ values ranging from 1.47 to 2.40 μL L^−1^, while *trans*-pinocarveol obtained by *C. tetradenius* showed an LC_50_ ranging between 1.40 and 1.75 μL L^−1^; *A. anethoides* possessed contact and fumigant toxicities against *T. castaneum* adults (LD_50_ = 28.80 μg adult^−1^ and LC_50_ = 13.05 mg L^−1^ air, respectively) and against *L. serricorne* (LD_50_ = 24.03 μg adult^−1^ and LD_50_ = 8.04 mg L^−1^ air, respectively), making them potentially suitable for pest management by reducing the use of synthetic insecticides.

The insecticidal action of *trans*-pinocarveol and pinocarvone could be linked to their chemical structure. α,β-Unsaturated compounds are reported to be alkylating agents with mutagenic properties [43]. *trans*-Pinocarveol is an α, β-unsaturated alcohol, while pinocarvone belongs to the chemical class of *α,β*-unsaturated ketones, which are well-known alkylating compounds with marked toxicity, as are myrtenal, verbenone, and carvone. The *α,β*-unsaturation is responsible for improved polarizability of the ketone moiety, which leads to enhanced London dispersion-type intermolecular attractive forces [44]. These structure-related properties could be the basis of the insecticidal/acaricidal mode of action. Additional studies on these two compounds should be performed.

Further efforts to evaluate possible non-target effects of this EO on useful arthropods [45,46] as well as to improve the field stability and effectiveness of the tested botanical product through its formulation in micro- or nanoemulsions are still needed [45,47,48,49,50].

## 3. Material and Methods

### 3.1. Plant Material and Essential Oil Preparation

Aerial parts of *M. moschatus* were collected in September 2019 at Ifandàna, Madagascar (S 22°00′39″, E 46°23′03″). Identification of plant was performed by one of us (R.R.) at the Parc Botanique et Zoologique de Tsimbazaza, Antananarivo. The voucher specimen (code RR-0002) was deposited at the Herbarium of the Parc Botanique et Zoologique de Tsimbazaza and the Centre National d’Application des Recherches Pharmaceutiques. The EO, of transparent color, was obtained in 0.02% (*w*/*w* f.w.) from freshly collected aerial parts of *M. moschatus* after 3 h of steam distillation. It was stored at +4 °C until use.

Furthermore, a botanical insecticide approved for organic farming in the Czech Republic was tested as positive control on the various arthropod species mentioned above. The insecticide’s commercial name was Rock Effect (Agro CS a.s., Česká Skalice, Czech Republic) and it is a commercial formulation of *P. pinnata* oil. The oil content is declared as 868.5 g·L^−1^. It is generally used as a 1 to 3% aqueous solution.

### 3.2. Gas Chromatography–Mass Spectrometry (GC–MS) Analysis

The chemical composition of the *M. moschatus* EO was determined using an Agilent 6890 N gas chromatograph equipped with a single quadrupole 5973 N mass spectrometer and an auto-sampler 7863 (Agilent, Wilmington, DE). The separation of the compounds was achieved with an HP-5 MS capillary column (30 m, 0.25 μm i.d., 0.1 μm f.t.; 5% phenylmethylpolysiloxane), provided by Agilent (Folsom, CA, USA). The conditions of the analysis and the identification method for each compound were the same as that reported by Pavela et al. [51].

### 3.3. Insecticidal and Acaricidal Experiments

#### 3.3.1. Target Organisms

*Metopolophium dirhodum* aphids, *S. littoralis* moths, *T. urticae* mites, *Cx. quinquefasciatus,* and *M. domestica* were tested. These species have been reared under controlled laboratory conditions for more than 20 generations. The following uniform individuals were selected for the experiments: *M. dirhodum* adults (1–2 days old), *S. littoralis* larvae (3rd instar, larval weight 10.2 ± 3.1 mg), *Cx. quinquefasciatus* larvae (early 3rd instar), *M. domestica* adults (female, 2–5 days old), and *T. urticae adults* (2–3 days old).

Arthropod mass-rearing in brief: *M. dirhodum* was reared on wheat plants in pots with a diameter of 10 cm; *S. littoralis* was reared on a semi-synthetic diet developed in the CRI laboratory that was based on soybean meal, agar, and vitamins; adults were fed honey solution and allowed to mate and lay eggs on prepared filter paper; *T. urticae* was reared on beans (*Phaseolus vulgaris* L.) planted in 10 cm diameter pots filled with a common garden substrate, and placed in a growth chamber [51]; larvae of *Cx. quinquefasciatus* were fed dried dog biscuits, adults were allowed to mate, and the females were given blood so that they could complete the development of the eggs, which they then laid in prepared containers of water; larvae of *M. domestica* were reared on a semi-synthetic diet developed at CRI consisting of a mixture of milk, agar, and sawdust; adults were fed powdered milk and sugar solution, eggs were laid on cotton wool dipped in sweet milk.

All arthropod species were maintained at 25 ± 1 °C, 70 ± 3% R.H., and 16:8 h (L:D). All experiments described below were performed under the same conditions.

#### 3.3.2. Insecticidal and Acaricidal Activity

The EO was dissolved in acetone to obtain a series of concentrations that, when applied at 1 µL, gave the following doses: for houseflies 10, 20, 30, 50, 80, and 100 µg adult^−1^; for moth larvae 20, 30, 40, 50, 60, and 80 µg larva^−1^. Before application, the arthropods were immobilized using CO_2_. As a negative control, acetone alone (Sigma-Aldrich, Czech Republic) was used in the experiments. After application, the adults and larvae were transferred to rearing containers with dimensions of 15 × 12 × 8 cm with a perforated lid, and the usual food. Twenty individuals were used for each repetition, and the experiment was repeated 4 times.

In tests targeting larvae of *Cx. quinquefasciatus,* EO was mixed in dimethyl sulfoxide (DMSO, Sigma-Aldrich, Czech Republic) and tested according to WHO (1996) with minor modifications by Pavela and Sedlák [52]. Each time, 1 mL of DMSO, containing a defined amount of EO, was mixed in 99 mL of water, and a concentration series containing 20, 40, 60, 80, and 100 mg mL^−1^ of EO was prepared. DMSO alone was used as a negative control. For each replicate, 20 larvae were used, and the experiment was repeated 4 times.

In tests targeting *M. dirhodum* and *T. urticae*, EO was mixed in Tween 80 (Sigma-Aldrich, Czech Republic) in a 1:1 (v:v) ratio. Subsequently, a concentration series was prepared (1.5, 2.0, and 3.0, 4.0, and 5.0 mL L^−1^, or 0.5, 1.0, 1.5, 2.0, and 3.0 mL L^−1^ for *M. dirhodum* and *T. urticae*, respectively) by thoroughly mixing the modified EO in water. Adults of *M. dirhodum* were transferred to wheat leaves using a fine brush (20 adults per plant in a 9 cm diameter pot); *T. urticae* adults were also transferred with a fine brush to bean leaves (20 adults per plant in a 9 cm diameter pot). The plants were sprayed using an electric applicator (5 mL of solution per plant). The experiment was replicated 5 times. For all tested species, the positive control was the commercial pesticide Rock Effect, a.i. *P. pinnata* oil.

Arthropod mortality was assessed 24 h after treatment. Mortality was corrected using Abbott’s formula [53] (Abbott 1925); LD_50_(_90_) and LC_50_(_90_) were estimated by probit analysis [54].

## 4. Conclusions

The findings of our work may open a new perspective on the use of *M. moschatus* in Madagascar as a source of botanical insecticide and acaricide ingredients. One of the advantages of this plant species is its ease of cultivation and growth in arid and semi-arid areas, offering the possibility to extract its EO in both dry and wet seasons. Thus, following the example of pyrethrum [55], *M. moschatus* may represent a new non-food cash crop to produce pesticidal products by Malagasy smallholder farmers. A future challenge born from this work is to prepare an artificial blend made up of the main EO constituents, such as *trans*-pinocarveol and pinocarvone, and to test it in insecticidal and acaricidal assays. However, difficulty in supplying these compounds may limit this applied research. Therefore, new synthetic or extractive approaches for these compounds should be pursued to have high disposability of these chemicals for agrochemical purposes.

## Figures and Tables

**Table 1:** Chemical composition of the *Myrothamnus moschatus* essential oil.

No.	Component ^a^	RI ^b^	RI Lit. ^c^	% ^d^	ID Method ^e^
1	*α*-pinene	926	932	8.8 ± 1.2	Std
2	camphene	939	946	0.4 ± 0.1	Std
3	thuja-2,4(10)-diene	944	953	0.1 ± 0.0	RI,MS
4	benzaldehyde	956	952	Tr ^f^	Std
5	*β*-pinene	967	974	0.6 ± 0.2	Std
6	*p*-cymene	1020	1020	1.8 ± 0.4	Std
7	limonene	1024	1024	0.8 ± 0.2	Std
8	1,8-cineole	1026	1026	0.7 ± 0.2	Std
9	*γ*-terpinene	1055	1054	Tr	Std
10	*p*-cymenene	1086	1089	0.1 ± 0.0	RI,MS
11	6-camphenone	1092	1095	0.1 ± 0.0	RI,MS
12	*trans*-*p*-mentha-2,8-dien-1-ol	1118	119	0.3 ± 0.0	RI,MS
13	α-campholenal	1122	1122	0.5 ± 0.1	RI,MS
14	*trans*-pinocarveol	1137	1135	37.7 ± 4.2	Std
15	*trans*-pinocamphone	1157	1158	0.1 ± 0.0	RI,MS
16	pinocarvone	1159	1160	20.8 ± 3.1	RI,MS
17	*p*-mentha-1,5-dien-8-ol	1164	1166	Tr	RI,MS
18	*cis*-pinocamphone	1168	1172	Tr	RI,MS
19	*p*-cymen-8-ol	1184	1179	0.5 ± 0.2	RI,MS
20	*trans*-*p*-mentha-1(7),8-dien-2-ol	1184	1187	2.2 ± 0.4	RI,MS
21	myrtenal	1189	1194	1.8 ± 0.3	Std
22	myrtenol	1191	1194	1.5 ± 0.3	Std
23	*cis*-piperitol	1196	1195	0.3 ± 0.1	RI,MS
24	*trans*-carveol	1215	1215	0.7 ± 0.1	RI,MS
25	4-methylene	1216	1216	0.5 ± 0.1	RI,MS
26	*cis*-*p*-mentha-1(7),8-dien-2-ol	1224	1227	1.4 ± 0.3	RI,MS
27	carvone	1239	1239	0.2 ± 0.0	Std
28	perilla aldehyde	1268	1269	0.1 ± 0.0	RI,MS
29	bornyl acetate	1280	1287	0.2 ± 0.1	Std
30	*trans*-pinocarvyl acetate	1295	1298	0.1 ± 0.0	RI,MS
31	myrtenyl acetate	1321	1324	0.5 ± 0.1	RI,MS
32	perillyl acetate	1433	1436	4.9 ± 0.9	RI,MS
33	*β*-selinene	1476	1489	10.2 ± 1.3	RI,MS
34	α-selinene	1481	1498	0.1 ± 0.0	RI,MS
					
	Total identified (%)			98.2	
	Chemical classes (%)				
	Monoterpene hydrocarbons			12.6	
	Oxygenated monoterpenes			75.3	
	Sesquiterpene hydrocarbons			10.2	
	Others			Tr	

^a^ Components were eluted from an HP-5MS column (30 m l. × 0.25 mm i.d., 0.1 μm f.t.). ^b^ Linear retention index experimentally determined with respect to a mixture of C_7_-C_30_ *n*-alkanes (Sigma-Aldrich) according to the Van den Dool and Kratz formula [27]. ^c^ Retention index value taken from ADAMS or FFNSC3 libraries. ^d^ Peak area relative percentages are the mean of two independent injections ± SD. ^e^ Method of identification: Std, RT, RI, and MS overlapping with analytical standard (Sigma-Aldrich); RI, coherence of the calculated RI with those stored in ADAMS and FFNSC3 libraries. MS, matching of mass fragmentation with that of spectra contained in ADAMS, NIST17, FFNSC3, and WILEY275 libraries. ^f^ Traces, % < 0.1.

**Table 2:** Acute toxicity of the *Myrothamnus moschatus* essential oil on selected insect and mite species.

Arthropod Species and Instar	Unit	LD_50_	CI_95_ ^a^	LC_90_	CI_95_ ^b^	*χ*^2^ (*d.f.*)	*p*-Value
*Musca domestica* adult	µg adult^−1^	22.7	17.4–30.3	109.6	75.7–175.8	1.545 (4)	0.818 ns
*Spodoptera littoralis* larva	µg larva^−1^	35.6	30.1–41.3	79.2	66.1–101.3	0.933 (4)	0.837 ns
*Culex quinquefasciatus* larva	µg mL^−1^	43.6	37.2–50.8	111.4	90.5–149.5	0.750 (3)	0.212 ns
*Metopolophium dirhodum* adult	mL L^−1^	2.4	2.2–2.6	5.8	5.5–7.1	1.196 (3)	0.753 ns
*Tetranychus urticae* adult	mL L^−1^	1.2	0.9–1.7	3.3	2.3–8.7	2.703 (3)	0.439 ns

^a^ 95% confidence interval relative to LD_50_ values. ^b^ 95% confidence interval relative to LC_50_ values. ns = not significant (*p* > 0.05).

**Table 3:** Acute toxicity of the positive control, a commercial botanical insecticide based on *Pongamia pinnata* oil, on selected insect and mite species.

Arthropod Species and Instar	Unit	LD_50_	CI_95_ ^a^	LC_90_	CI_95_ ^b^	*χ*^2^ (*d.f.*)	*p*-Value
*Musca domestica* adult	µg adult^−1^	˃500	-	-	-	-	-
*Spodoptera littoralis* larva	µg larva^−1^	18.2	15.6–19.8	28.6	26.8–32.5	2.451 (3)	0.751 ns
*Culex quinquefasciatus* larva	µg mL^−1^	275.4	256.8–321.7	1285.7	1211.5–1354.4	3.512 (3)	0.425 ns
*Metopolophium dirhodum* adult	mL L^−1^	12.5	9.6–13.8	21.7	18.6–22.3	2.525 (3)	0.598 ns
*Tetranychus urticae* adult	mL L^−1^	5.8	5.1–6–8	10.1	9.2–12.7	1.851 (3)	0.845 ns

^a^ 95% confidence interval relative to LD_50_ values. ^b^ 95% confidence interval relative to LC_50_ values. ns = not significant (*p* > 0.05).

## Data Availability

Data are contained within the article.
